# Bronchoscopy Laser and Silicone Y-Stents as Emergency Airway Management in Central Airway Stenosis Due to Secondary Thyroid Carcinoma: A Case Report

**DOI:** 10.1155/2022/6338073

**Published:** 2022-11-07

**Authors:** Mia Elhidsi, Dicky Soehardiman, Agung Wibawanto, Haris Maruli, Maryastuti, Prasenohadi, Elisna Syahruddin

**Affiliations:** ^1^Department of Pulmonology and Respiratory Medicine, Faculty of Medicine, Universitas Indonesia, Persahabatan National Respiratory Referral Hospital, Jakarta, Indonesia; ^2^Indonesian Bronchoscopy Society, Perbronki, Jakarta, Indonesia; ^3^Department of Thoracic, Cardiac, and Vascular Surgery, Persahabatan National Respiratory Referral Hospital, Jakarta, Indonesia; ^4^Department of Surgery, Persahabatan National Respiratory Referral Hospital, Jakarta, Indonesia; ^5^Department of Radiology, Persahabatan National Respiratory Referral Hospital, Jakarta, Indonesia

## Abstract

Total airway obstruction in thyroid cancer is rare and has high morbidity and mortality. Airway management in such cases is challenging, especially in cases in which thyroid masses cannot be totally resected. It is important to choose the appropriate airway treatment modality. Currently, therapeutic rigid bronchoscopy procedures and endoluminal lasers, as well as airway stent insertion, are a management modality of near-total malignant airway obstruction. We report a rigid bronchoscopy procedure combined with laser and Y-stent silicone insertion in thyroid cancer with extension infiltration, as well as compression in the trachea covering the subglottic tracheal area up to the main carina and tracheo-bronchomalacia, manifesting as acute respiratory failure.

## 1. Introduction

Total airway obstruction in thyroid cancer is rare, with an incidence rate of about 6%–14% of cases [1]. Thyroid mass can be the cause of total airway obstruction through several mechanisms: local invasion, extrinsic tumour compression, or a combination of both [2]. Total airway obstruction caused by thyroid mass can be asymptomatic to life-threatening. Airway management in such cases is challenging, especially in cases in which thyroid masses cannot be totally resected. Currently, therapeutic rigid bronchoscopy procedures and endoluminal lasers, as well as airway stent insertion, are a management modality of near-total malignant airway obstruction [3, 4]. The use of silicone stents has been reported to overcome airway obstruction in cases of advanced compressive tracheal tumours [5]. We report a rigid bronchoscopy procedure combined with laser and Y-stent silicon insertion in thyroid cancer with extension infiltration, as well as compression in the trachea covering the subglottic tracheal area up to the main carina, manifesting as acute respiratory failure.

## 2. Case Presentation

A 50-year-old Javanese male patient came to the emergency room with severe shortness of breath for 10 days, accompanied by breath sounds. The patient complained of a lump growing on the neck for 10 years. Physical examination revealed 85% oxygen saturation on 15 L/min non-rebreather mask oxygen supplementation, accompanied by increased work of breathing, respiratory rate 30x/min, tachycardia 110x/min, and blood pressure 140/90 mmHg. An immobile mass on the right thyroid was palpable; the size was 3 × 2 × 1 cm with hard consistency. An immobile mass on the left thyroid was 3 × 2 × 1 cm with hard consistency.

A thoracic and neck-computed tomography (CT) scan 2 weeks before the patient's admission to the emergency room indicated a solid mass in the right neck, suggesting lymphadenopathy, soft tissue mass, or soft tissue metastases. It was possibly a primary metastatic thyroid mass, superior mediastinal mass, or bilateral enlargement of the cervical lymph nodes. Electromyography examination with the Harvey–Masland test suggested the presence of myasthenia gravis.

**Figure 1:**
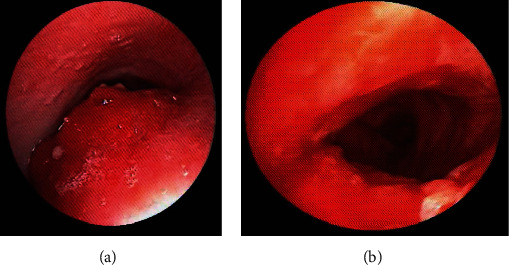
Bronchoscopic findings before and after laser and rigid scope mass removal. (a) Infiltrative and compression thyroid mass obstructing the tracheal lumen. The distal lesion cannot be seen in this view. (b) Trachea after laser and rigid scope mass removal.

The bronchoscopy findings showed an infiltrative mass, a 2 cm protruding mass completely obstructing the proximal trachea 1.5 cm below the vocal cords, an infiltrative mass, and a 1.5 cm protruding mass closing the midtrachea 3.5 cm above the carina. Laser ablation through rigid bronchoscopy was then performed. The tracheal lumen was opened >50% posttreatment (Figure 1). The patient was stable and relieved of shortness of breath. Histopathological results of the distal tracheal biopsy indicated classical variant papillary thyroid carcinoma and tall cells.

**Figure 2:**
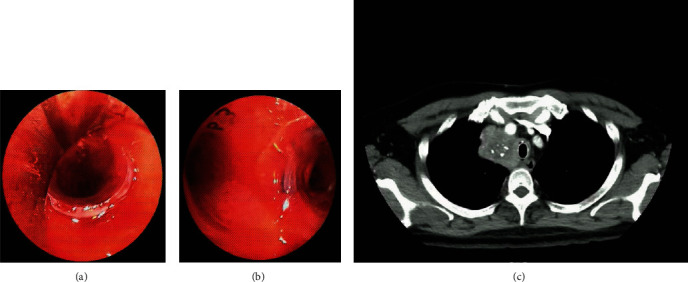
Bronchoscopy and thoracic-computerized tomography view of silicone Y-stent insertion. (a) Bronchoscopy view of silicone Y-stent at the subglottic trachea. (b) Bronchoscopy view of silicone Y-stent at the main carina. (c) Axial-computerized tomography view of airway stent at trachea. Arrow shows the airway stent; dotted arrow shows the thyroid mass.

Thyroidectomy by surgical oncology was performed 2 days after bronchoscopy. The right and left lobes of the thyroid appeared enlarged, dense, and tightly attached to the trachea and surrounding muscles. Tumour resection was performed in the left lobe and isthmus, leaving tumours in the right lobe and intrathorax. Two days after surgery, the patient experienced unconsciousness, severe dyspnoea, and stridor. An emergency bronchoscopy was performed and found compressive stenosis that obstructed >50% of the lumen starting from the carina up to 10 cm above it, accompanied by trachea-bronchomalacia. A silicone DUMON™ Y-stent 14 × 10 × 10 mm was implanted in 3 cm of the left main bronchus, 1 cm of the right main bronchus, and 10 cm of the trachea through a rigid bronchoscope (Figure 2).

After stent installation, the patient was extubated with stable respiration and hemodynamics, and there was no dyspnoea or abnormal breath sounds. Thoracic CT showed that the stent patented the airway (Figure 2). The patient was scheduled for thyroid ablation. A bronchoscopy evaluation is planned if there are respiratory complaints.

## 3. Discussion

A tumour protruding into the central airway is associated with a poor prognosis and is the most common cause of death in thyroid tumour cases [6, 7]. Complete resection with a negative margin is certainly the ideal treatment for localized advanced thyroid cancer. However, the large tumour size in this case and its attachment to the surrounding organs complicated the surgical procedure and left part of the thyroid mass. Surgical treatment of thyroid cancer recurrently involving the laryngeal nerve, trachea, and oesophagus is associated with high morbidity and risk of malacia [6–9]. Stent placement is not currently a standard procedure for postthyroidectomy surgery, although bronchoscopy can be used in some cases to evaluate the trachea before extubation to assess the malacia [8].

Emergency tracheostomy for airway management can be challenging. It could not be chosen in this case, since the tumour was infiltrating near the main carina, and there was trachea-bronchomalacia. Long stenosis from the subglottis to the carina and the presence of a large tumour mass made it difficult for the clinician to identify the structure of the trachea and its lumen. In addition, it was not easy to fix the tracheostomy tube due to the distance between the skin and the trachea [10]. Holting et al. found that tracheostomy procedures performed in cases of respiratory emergencies in patients with anaplastic thyroid carcinoma had a low survival rate. It is associated with a poor prognosis and delays in subsequent therapy due to complications of the tracheostomy procedure [11, 12].

Intraluminal mass ablation with laser bronchoscopy was chosen since it has sufficient power to vaporize tissues and has a coagulation effect [13]. Previous studies showed that the combination of rigid bronchoscopy and laser is a successful treatment for mixed types of intraluminal tumour growth and extraluminal tumour compression [14–16]. The laser procedure is relatively safe, with the most common complications being bleeding and perforation [17]. In our case, the laser target lesion was an infiltrative lesion, predominantly in the right lateral trachea. Laser application to the posterior side of the membranacea had to be avoided or performed carefully to prevent esophageal perforation and fistula formation.

At present, no high-quality studies have compared laser treatment with the hot method. In general, some factors, such as method selection, location, tumour characteristics, competencies, tool ability, extrinsic versus intrinsic compression level, and local expertise, are individual factors for consideration in each patient. Among the available modalities, only laser resection, argon plasma coagulation, and electrocautery procedures can destroy tissue sufficiently rapidly. Furthermore, the laser resection procedure has the best debulking capacity and may be as efficient as an argon plasma coagulation (APC) procedure in achieving hemostasis. At our center, the utilization of the laser is also a more familiar procedure, and the laser procedure provided fast and safe outcomes for this patient.

Although there was clinical improvement after the bronchoscopy mass removal procedure, the patient continued to experience airway obstruction due to tracheomalacia. Hence, a therapeutic bronchoscopy procedure was necessary, and in this situation, stent insertion was the best option. Any consideration of stent insertion must be based on the risks and benefits and must have patient consent. Experts recommend stent stabilization in severe tracheobroncho-malacia cases, and stent insertion was also a life-saving procedure in our case, as our patient had impaired consciousness and decreased oxygen saturation. In stable and mild-moderate dyspnoea patients, a lung function examination and the administration of continuous positive airway pressure (CPAP) ventilation can be performed [9].

The silicone stent is more widely used than the metallic stent in central airway obstruction. Sehgal et al. found that mounting a silicone Y-stent is considered safe and effective in managing airways involving the lower trachea and carina. Furthermore, the insertion procedure of a silicone Y-stent is relatively easier and has fewer complications compared to metallic types [18]. Aktas et al. found that some complications due to silicone Y-stent insertion are mucostasis, migration, and tissue growth at the edges of the stent. These complications can be managed to restore stent function. However, if not possible, the stents can be removed from the airway [19]. Moreover, after inserting the stents, the patient is eligible to undergo definitive therapy for thyroid cancer, with improvement in symptoms and quality of life [20].

## 4. Conclusion

Laser bronchoscopy ablation, followed by the installation of a silicon Y tracheobronchial stent, can be a suitable option for managing thyroid cancer with central airway obstruction due to mass infiltration and bronchomalacia with acute respiratory failure.

## Data Availability

All data generated during this study are included in this published article.
